# Effects of Zhishi Daozhi Decoction on the intestinal flora of nonalcoholic fatty liver disease mice induced by a high-fat diet

**DOI:** 10.3389/fcimb.2022.1005318

**Published:** 2023-01-04

**Authors:** Chao-Ran Bi, Jia-Tong Sun, Jian Du, Li-Yuan Chu, Yi-Jing Li, Xiao-Yu Jia, Yuan Liu, Wen-Ping Zhang, Yu-Chun Li, Yan-Jing Liu

**Affiliations:** ^1^ College of Traditional Chinese Medicine, Changchun University of Chinese Medicine, Changchun, China; ^2^ Department of Endocrinology, Metabolism and Gastroenterology, Third Affiliated Clinical Hospital of Changchun University of Chinese Medicine, Changchun, China; ^3^ Department of Ophthalmology, China-Japan Friendship Hospital of Jilin University, Changchun, China; ^4^ Department of Spleen and Gastroenterology, Jilin Provincial Academy of Traditional Chinese Medicine, Changchun, China

**Keywords:** nonalcoholic fatty liver disease, intestinal flora, Zhishi Daozhi Decoction, high-fat diet, traditional Chinese medicine

## Abstract

**Background and aims:**

Nonalcoholic fatty liver disease (NAFLD) is the most common type of chronic liver disease with a high incidence, and the situation is not optimistic. Intestinal flora imbalance is strongly correlated with NAFLD pathogenesis. Zhishi Daozhi Decoction (ZDD) is a water decoction of the herbs used in the classical Chinese medicine prescription Zhishi Daozhi Pills. Zhishi Daozhi Pills has shown promising hepatoprotective and hypolipidemic properties, but its specific mechanism remains unclear.

**Methods:**

Mice were fed on a high fat-rich diet (HFD) for ten weeks, and then the animals were administrated ZDD through oral gavage for four weeks. The serum liver function and blood lipid indexes of the mice were then tested using an automatic biochemical analyzer. H&E and Oil Red O staining were used to observe the pathological conditions of mice liver tissue, and 16S rRNA sequencing technology was used to analyze the changes in intestinal flora of mice. The concentration of short-chain fatty acids (SCFAs) in the gut of mice was analyzed by gas chromatography-mass spectrometry (GC-MS). The expression of tight junction (TJ) proteins between ileal mucosal epithelial cells was analyzed using the immunofluorescence technique.

**Results:**

ZDD was found to reduce the bodyweight of NAFLD mice, reduce serum TG, CHO, ALT, and AST levels, reduce fat accumulation in liver tissue, make the structure of intestinal flora comparable to the control group, and increase the concentration of intestinal SCFAs. It was also found to increase the expression of TJ proteins such as occludin and ZO-1, making them comparable to the control group.

**Conclusions:**

ZDD has a therapeutic effect on NAFLD mice induced by HFD, which may act by optimizing the intestinal flora structure.

## 1 Introduction

The prevalence of nonalcoholic fatty liver disease (NAFLD) is on the rise, impacting an increasing number of individuals globally. As the most common type of chronic liver disease, it frequently co-occurs with obesity, hypertension, and type 2 diabetes developing metabolic syndrome and is also considered a hepatic manifestation of metabolic syndrome (Loomba et al., 2021). In recent years, the number of people with dyslipidemia has reached 40.4%, whereas the number of people with NAFLD accounts for 25.2%, with a higher incidence in the young population (Gao, 2018). According to the current research, the mainstream pathogenesis of NAFLD includes the “second hit” theory, the lipotoxicity theory, iron elements, genetic factors, bile acids, intestinal microbes, and other mainstream theories. However, there is increasing evidence that an imbalance in intestinal flora substantially correlates with NAFLD (Milosevic et al., 2019; Makri et al., 2020). Furthermore, the theory of the liver-gut axis has been gradually connected and confirmed by studies on the intestinal flora, intestinal short-chain fatty acids (SCFAs), and intestinal mucosal mechanical barrier (Albillos et al., 2020).

The intestinal microbiota comprises bacteria, viruses, fungi, and other microorganisms in the gut, where the bacteria account for 95% of the total microbial flora. Intestinal flora imbalance occurs when the stability and diversity of the intestinal bacterial community are destroyed (Yılmaz and Eren, 2017) and can affect the number and function of intestinal SCFAs (Carter and Karpen, 2007; Blaut, 2014). SCFAs being the major metabolite of intestinal flora, can promote intestinal epithelial proliferation and minimize the intestinal epithelial cells’ apoptosis, thereby increasing the expression of tight junction (TJ) proteins to reduce intestinal mucosa permeability (Gui and Shen, 2016; Zhao et al., 2018). Consequently, when the number and function of SCFAs are damaged, the intestinal mucosal barrier is destroyed, increasing its permeability and allowing a large number of bacteria, their metabolites, cytokines, and other substances to enter the liver through the portal vein, bypassing the processing of the intrahepatic mononuclear macrophage system. This triggers a cascade of cytokines, resulting in an uncontrolled immune response and releasing many inflammatory mediators, aggravating liver damage and disease progression (Compare et al., 2012). This series of immune-inflammatory reactions ultimately lead to NAFLD’s occurrence and progression. This mechanism is also supported by the concept of the “gut-liver axis” proposed by Marshall (Marshall, 1998) in 1998. Furthermore, a study also reported that the transplantation of intestinal bacterial feces from obese mice to pseudo-germ-free mice could increase their weight and liver fat content, indicating that intestinal flora imbalance is an essential factor leading to NAFLD (Delaune et al., 2018).

NAFLD pathogenesis results from multiple factors; thus, treatment methods for this disease are numerous, including removing the cause, adjusting the diet structure and lifestyle, drug treatment, etc., yet no specific drug is available having an ideal curative effect on this disease. Zhishi Daozhi Pill comes from *Differentiation on Endogenous and Exogenous Diseases*, written by Dong-yuan Li in 1247. It is composed of eight different types of Chinese herbal medicine, including Zhishi (*Citrus aurantium*), Dahuang (*Rheum palmatum*), Huanglian (*Coptis chinensis*), Huangqin (*Scutellaria baicalensis*), Shenqu (*Massa Medicata Fermentata*), Baizhu (*Atractylodes macrocephala*), Fuling (*Poria cocos*), and Zexie (*Alisma orientate*). This Traditional Chinese medicine (TCM) prescription has the function of “eliminating stagnation, clearing dampness and heat”, which means it can act as a laxative and removes excess energy from the body. According to TCM theory, NAFLD is caused by “damp heat and food accumulation”, so there is sufficient evidence to use this prescription. Zhishi Daozhi Decoction (ZDD) is a water decoction of the herbs in the classical Chinese medicine prescription Zhishi Daozhi Pills, which shows satisfactory hepato-protecting and lipid-lowering effects (Liu, 2006; Zhou, 2010). However, the exact mechanism remains unknown. Therefore, this study aimed to investigate the impact and mechanism of ZDD on the NAFLD mice model through serological testing, pathological examination, and effects on the intestinal flora, intestinal SCFAs, and TJ proteins between ileal mucosal epithelial cells of NAFLD to verify its efficacy.

## 2 Materials and methods

### 2.1 Zhishi Daozhi Decoction preparation

The ZDD Chinese herbal medicines required for this experiment for four weeks include 12.8 g of Citrus aurantium (Qingjiang County, Jiangxi Province), 6.4 g of Rheum palmatum (Pingwu County, Sichuan Province), 19.2 g of Coptis chinensis (Dayi County, Sichuan Province), 12.8 g of Scutellaria baicalensis (Ba County, Sichuan Province), 19.2 g of Massa Medicata Fermentata (Dehua County, Fujian Province), 19.2 g of Atractylodes macrocephala (Xinchang County, Zhejiang Province), 19.2 g of Poria cocos (Shuangbai County, Yunnan Province), and 12.8 g of Alisma orientate (Pucheng County, Fujian Province), and were purchased from the Decoction Pieces Pharmacy of the Third Affiliated Clinical Hospital of the Changchun University of Chinese Medicine. Herbs were obtained from qualified suppliers on the basis of standards specified in the Chinese Pharmacopoeia (2020 Edition). Put the above Chinese herbal medicines into a porcelain jar, add 300 mL of distilled water, boil for 30 min, fifilter, collect the first fifiltrate, add 200 mL of distilled water and continue to boil for 30 min, fifilter, combine the two fifiltrates, concentrate into a raw drug concentration of 1.45 g/mL of ZDD, store at -4°C for backup. Similarly, polyene phosphatidylcholine (PPC) was purchased (National Medicine Permit No. H20059010, Sanofi-Aventis, Beijing) and was dissolved in distilled water to prepare a solution with a concentration of 12 mg/mL.

### 2.2 Animals and treatment

A total of 46 7-week-old male C57BL/6 mice (20g ± 2 g) were provided by Jilin Qianhe Model Biotechnology Limited Company (Experimental Animal License No. SYXK (Ji) 2019-0012). The study was approved by the Animal Experiment Ethics Committee of the First Hospital of Jilin University (Approval Number 20210627). The mice were raised in the National Local Joint Engineering Laboratories of Animal Models for Human Diseases. After one week of adaptive feeding, 46 mice were randomly divided into two groups: The Control group (n=13) and HFD group (n=33). The control group (C group) mice were fed 10% energy fat-supplied purified feed (Product ID XTCON50J). HFD group was fed 60% energy fat-supplied purified feed (Product ID XTHF60). The above-mentioned feeds were purchased from Jiangsu Xietong Pharmaceutical Bioengineering Limited Company. After the 10th week of feeding, three mice from each group were randomly selected for serological and pathological tests to confirm the successful disease model. Mice in the HFD group were randomly divided into Model group (M group) (n=10), PPC group (P group) (n=10), and ZDD group (Z group) (n=10). C and M groups were given distilled water 0.1ml/10g/d by gavage; the P group was given PPC 120 mg/kg/d, and the Z group was given ZDD 14.5g/kg/d. The gavage volume of each group was 0.1 mL/10 g, and the treatment period of each group was four weeks.

The body weight of the mice was observed once a week during the experiment. Before the end of treatment, mice feces were collected for three consecutive days, placed in sterile EP tubes, and kept in a -80 °C refrigerator for detecting intestinal flora and SCFAs by 16S rRNA sequencing and gas chromatography-mass spectrometry (GC-MS). Following the last dose administration, all animals in all groups were put on fasting for 12 hours with free access to water only. The next day, all animals were anesthetized with an intraperitoneal injection of 10% chloral hydrate solution (0.35 mL/100 g) and administered at the dosage of 0.8 mL. Blood samples from each group was collected from the eyeballs for serological testing. Similarly, fresh samples from the same part of the left lobe of mice liver were taken from each group and placed in 4% paraformaldehyde fixative solution and OCT embedding solution for the pathological testing. Fresh ileal tissues of mice from each group were taken, contents removed, and placed in a 4% paraformaldehyde fixative solution for immunofluorescence detection.

### 2.3 Reagents and instruments

The reagents of triglyceride (TG), cholesterol (CHO), alanine aminotransferase (ALT), aspartate aminotransferase (AST), and glutamyl transpeptidase (GGT) were provided by Shenzhen Rayto Life Technology Company. Harrel’s hematoxylin staining solution, hematoxylin and eosin (H&E) staining differentiation solution, and water-soluble eosin (Y) staining solution were provided by Thermo Scientific. Oil Red O staining solution (BBI, 1320-06-5), Gel extraction kit (Qiagen, Germany), TruSeq^®^ DNA PCR-Free Sample Prep Kit (Illumina, USA), The chromatographic purity of acetic acid (AA), propionic acid (PA), isobutyric acid (IBA), butyric acid (BA), valeric acid (VA), caproic acid (CA), 2-methylvaleric acid, and methyl tert-butyl ether are CNW; the chromatographic purity of isovaleric acid (IVA) is Aladdin; occludin (Proteintech-66378-1-Ig); ZO-1 (Proteintech-21773-1-AP); FITC-labeled goat anti-mouse IgG (Abcam-b6785); Alexa Fluor 555-labeled goat anti-rabbit IgG (Invitrogen-A27039); Goat serum (Solarbio-SL038); DAPI (Solarbio-D8200); Anti-fluorescence decay mountant (Solarbio-S2100).

The equipment used was a low-temperature high-speed centrifuge (JOAN LAB-LC-12S), -80°C refrigerator (Thermo Scientific), fully automatic biochemical analyzer (Shenzhen Rayto Life Technology-Chemray 240/800), automatic tissue dehydrator (Thermo-ExcelsiorES), tissue embedding machine (Thermo-HistoStar), Semi-automatic rotary microtome (Thermo-HM340E), Cryostat (Thermo-HM550), Qubit@2.0 Fluorometer (Thermo Fisher Scientific, USA), Bioanalyzer (Agilent-2100, USA), GC-MS/MS (Agilent-7890B-7000D), Ball mill (Retsch-MM400), Electronic Balance (RADWAG-AS 60/220. R2), Multi-tube vortex shaker (Shanghai Jingxin-MIX-200, Electric heating constant temperature incubator (Tianjin Test-DH36001B), Ultrapure water system (HealForce-NW10LVF), and microscope (OLYMPUS-BX43).

### 2.4 Serological test

Whole blood serum separation was carried out at 6°C, 3000 rpm for 15 min TG, CHO, ALT, AST, and GGT indexes were tested by an automatic biochemical analyzer.

### 2.5 Pathological test

H&E staining: The liver tissue samples were embedded in wax blocks, cut into thin slices, and dewaxed. The sections were nuclear stained with hematoxylin solution, cytoplasm stained with eosin solution, followed by dehydration and sealing.

Oil red O staining: The liver tissues were frozen into blocks, cut into thin slices, and dried. The slices were immersed in isopropanol, dipped in oil red O staining solution, counterstained with hematoxylin staining solution, and sealed. All slides were then observed under an optical microscope.

### 2.6 16S rRNA sequencing

DNA samples were extracted by the CTAB method. The V3-V4 region in the extracted DNA was amplified by PCR using the forward primer 338F (5’-ACTCCTACGGGAGGCAGCAG-3’) and the reverse primer 806R (5’-GGACTACHVGGGTWTCTAAT-3’). The mixed PCR products were purified using Qiagen Gel Extraction Kit.

Sequencing libraries were generated using the TruSeq^®^ DNA PCR-No Sample Prep Kit. Library quality assessment was performed using Qubit@2.0 Fluorometer and an Agilent Bioanalyzer 2100 system. The library was sequenced on the Illumina NovaSeq platform.

Data quality control and analysis: Before bioinformatics operations, high quality target sequences have been obtained in advance. The specific steps involve splitting (FLASH V1.2.7: http://ccb.jhu.edu/software/FLASH/) (Magoč and Salzberg, 2011), filtering (QIME V1.9.1: https://qiime.org/scripts/splitlibrariesfastq.html) (Caporaso et al., 2010; Bokulich et al., 2012) and chimera removing (Silva database: https://www.arb-silva.de/and UCHIME Algorithm: http://www.drive5.com/usearch/manual/uchimealgo.html) (Edgar et al., 2011).

### 2.7 GC-MS

The acquisition conditions of chromatography and mass spectrometry are as follows: the injection volume is 2 μL; the Front Inlet Mode was 1:1, and the carrier gas was Helium. The column used was DB-FFAP (30 m x 0.25 mm x 0.25 µm); the column flow was 1.2 mL/min. The oven temperature ramp was held at 90°C for one min, then raised to 100°C at a rate of 25°C/min, then raised to 150°C at a rate of 20°C/min, held for 0.6 min, and raised to 200°C at a rate of 25°C/min, held for 0.5 min, after running for 3 min. The front injection temperature was 200°C, the transfer line temperature was 230°C, the ion source temperature was 230°C, and the quad temperature was 150°C.

### 2.8 Immunofluorescent detection

The ileum tissue was made into a wax block, cut into thin slices, and dewaxed. The antigen was recovered at a high temperature with antigen retrieval solution, and goat serum was added dropwise. Primary antibody and fluorescent secondary antibody were added dropwise; the dilution ratio of primary antibody occludin was 1:500, and the secondary antibody was FITC-labeled goat anti-mouse IgG (green light). Another primary antibody, ZO-1, was diluted at a ratio of 1:500, and the secondary antibody was Alexa Fluor 555-labeled goat anti-rabbit IgG (red light). For nuclear staining, DAPI was added dropwise. Anti-fluorescence quencher was added dropwise. The slides were covered and observed under a fluorescence microscope. Image-ProPlus 6.0 software was used for analysis.

### 2.9 Statistical analysis

SPSS 26.0 statistical software was used for data analysis. The T-test was used to compare measurement data between two groups. The data were expressed as mean ± standard deviation (SD) if they conformed to the normal distribution. The Mann-Whitney U rank-sum test was used if they did not conform to the normal distribution, and it was expressed as the median. If the measurement data compared between multiple groups conformed to the normal distribution, it was analyzed by one-way ANOVA. The Kruskal Wallis H test was used if they did not conform to the normal distribution. Graph Pad Prime 8 was used for drawing, and P<0.05 indicates a statistical difference.

## 3 Results

### 3.1 Therapeutic effect of ZDD on NAFLD model mice

**Figure 1:**
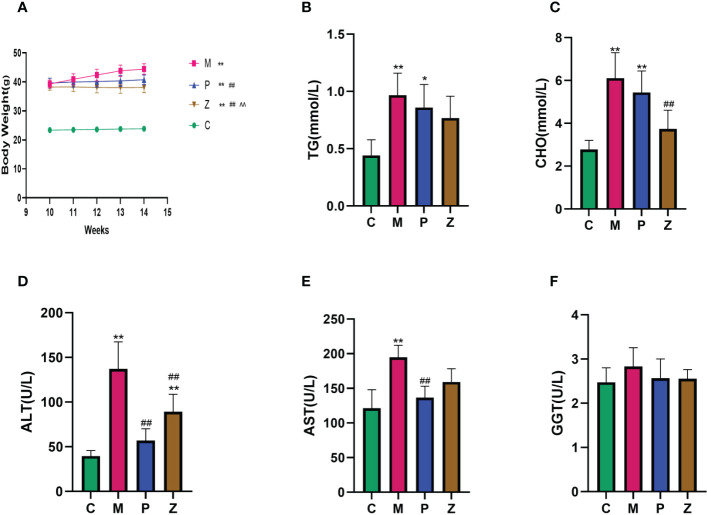
Comparison of Body Weight and Biochemical Assays in each group. **(A)** Body Weight. **(B)** Serum TG. **(C)** Serum CHO. **(D)** Serum ALT. **(E)** Serum AST. **(F)** Serum GGT. The above data are expressed as mean ±SD. * is P<0.05 compared with C group; ** is P<0.01 compared with C group; # is P<0.05 compared with M group; ## is P<0.01 compared with M group; ^ is P<0.05 compared with P group; ^^ is P<0.01 compared with P group.

There was a significant difference in body weight between the HFD group and the C group before the decoction intervention, indicating that the modeling was successful. After four weeks of treatment, both P and Z groups could effectively prevent weight gain, with the Z group showing a better effect (**Figure 1A**). In terms of blood lipids (**Figures 1B, C**), there is a significant difference between the C and M groups. Although both the P and Z groups can reduce blood lipid, the Z group performs better in CHO index. In terms of serum liver function (**Figures 1D, E**), there is a significant difference between the C and M groups. Although both the P and Z groups can reduce liver function, the P group performs better in ALT and AST indexes. In addition, there is no significant difference among the groups in the GGT index of liver function (**Figure 1F**).

**Figure 2:**
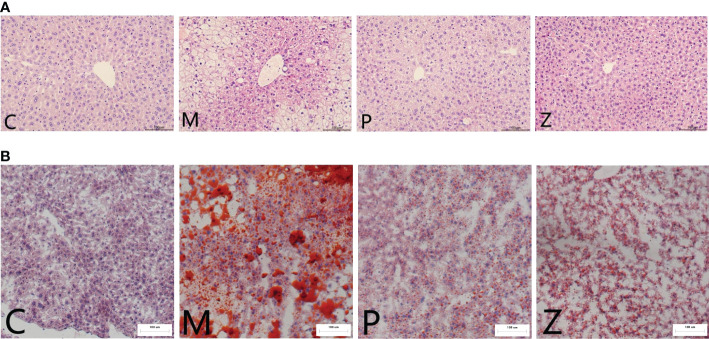
Comparison of Histopathological Examination in each group. **(A)** H&E staining of mice liver tissue (light microscope 200 ×). **(B)** Oil red O staining of mice liver tissue (light microscope 200 ×).

Furthermore, in terms of H&E staining, the structure of hepatic lobules of mice in the M group was disordered, as was the arrangement of hepatocytes, which were swollen, filled with fat vacuoles of varying sizes, and the nuclei were marginalized. However, the condition of the P and Z groups was significantly better than that of the M group (**Figure 2A**). Oil Red O staining revealed several red lipid droplets of different sizes in the hepatocytes of mice in the M group. The cells were irregular in shape with unclear borders and fused into sheets, and the nuclei were marginalized. However, the condition of the P and Z groups was significantly better than that of the M group (**Figure 2B**). The PPC and ZDD both produced therapeutic effects on the NAFLD mice model, with each exerting differently emphasizing effects.

### 3.2 The effect of ZDD on the intestinal flora of NAFLD model mice

#### 3.2.1 Analysis of the intestinal flora diversity

**Figure 3:**
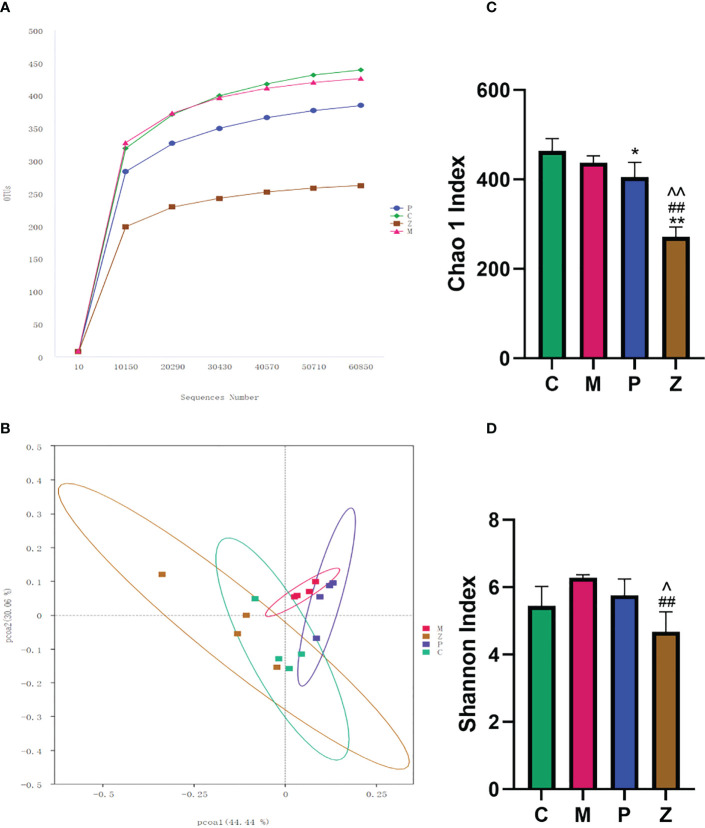
Intestinal flora diversity analysis. **(A)** Rarefaction Curve. The abscissa is the number of sequencing strips randomly selected from a sample, and the ordinate is the number of OTUs that can be constructed based on the number of sequencing strips used to reflect the sequencing depth. Curves with different colors represent different samples. **(B)** PCOA chart. The abscissa and ordinate axes are the two selected principal axes. The percentages indicate the degree of interpretation of the differences in the composition of samples by the principal coordinates. The scales on the two principal axes are relative distances, which have no practical significance: samples in different groups are distinguished by their respective colors, and the closer the point-to-point distance between samples is, the more similar the flora composition between samples is. **(C)** Chao 1 Index indicates community richness. **(D)** Shannon Index shows community diversity. Data in histograms are expressed as Mean+SD. * is P<0.05 compared with C group: ** is P<0.01 compared with C group: # is P<0.05 compared with M group: ## is P<0.01 compared with M group: ^ is P<0.05 compared with P group: ^^is P<0.01 compared with P group.

The rarefaction curve indicated that the sequencing quality of this sample was satisfactory for subsequent analysis (**Figure 3A**). Both P and Z groups showed a decreasing trend in community richness, whereas both C and M groups were significantly different from the Z group (**Figure 3C**). Furthermore, the Z group showed a decreasing trend in terms of community diversity. The Z group was significantly different from the M group, while there was no significant difference between the Z and C groups (**Figure 3D**).

Principal Component Analysis (PCoA) was used to investigate the similarity or difference in sample composition to determine differences in the composition of the intestinal flora of mice in each group. The results showed that each group of samples had better intra-group aggregation but differed from other sample groups (**Figure 3B**). The differences between the samples in each group were further evaluated using the Amova algorithm, and the results indicated samples in groups Z and C had no difference. Both C and Z groups can form a significant difference from the M group, but P group cannot form a significant difference from the M group. (**Table 1**). This demonstrates that the NAFLD model created by HFD can change the intestinal flora of mice, while ZDD can alter the intestinal flora of the mice to be more similar to that of healthy mice in the control group.

**Table 1:** Significant differences in the structure of the intestinal community of mice in each group (Amova).

*vs*-group	ss	df	MS	Fs	P-value
C-M	0.0700192 (0.0721107)	1 (6)	0.0700192 (0.0120185)	5.82598	0.018*
C-P	0.0737904 (0.0825321)	1 (6)	0.0737904 (0.0137553)	5.36449	0.052
C-Z	0.0712606 (0.179587)	1 (6)	0.0712606 (0.0299312)	2.38081	0.06
M-P	0.0153254 (0.0523039)	1 (6)	0.0153254 (0.00871732)	1.75804	0.094
M-Z	0.105141 (0.149326)	1 (6)	0.105141 (0.0248876)	4.22464	0.001**
P-Z	0.148381 (0.159775)	1 (6)	0.148381 (0.0266291)	5.57215	0.021*

SS is the total variance, also known as the sum of squared deviations; df is the degree of freedom; MS is the mean square (difference), i.e., SS/df; Fs is the F test value; p-value is P-value, less than 0.05 indicates between groups Significant difference. Inside the parentheses are the values corresponding to the residuals. * is P<0.05; ** is P<0.01.

#### 3.2.2 Analysis of the intestinal flora composition

**Figure 4:**
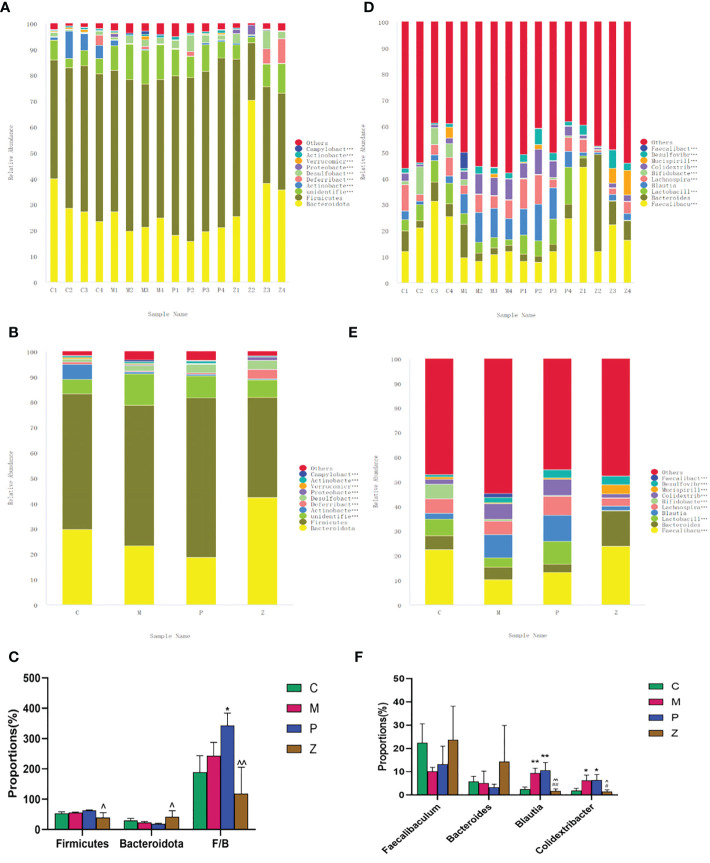
Analysis of the intestinal flora composition. **(A)** Percent of community abundance among sample on phylum level. **(B)** Percent of community abundance among groups on phylum level. **(C)** Histogram of differences in intestinal flora abundance on phylum level. **(D)** Percent of community abundance among sample on genus level. **(E)** Percent of community abundance among groups on genus level. **(F)** Histogram of differences in intestinal flora abundance on genus level. Data in histograms are expressed as mean+SD. * is P<0.05 compared with C group; ** is P<0.01 compared with C group: # is P<0.05 compared with M group; ## is P<0.01 compared with M group: ^ is P<0.05 compared with P group: ^^ is P<0.01 compared with P group.

At the phylum level, Firmicutes and Bacteroidota dominated the flora composition (**Figures 4A, B**). The former accounted for a relatively small proportion in the Z group, while the latter accounted for a rather large proportion. Some studies have described that the intestinal flora of obese animals and humans exhibits a higher Firmicutes/Bacteroidetes ratio compared with normal-weight individuals, proposing this ratio as an eventual biomarker (Zou et al., 2020; De Bandt et al., 2011). Considering the importance of Firmicutes and Bacteroidetes in metabolic diseases, the Firmicutes/Bacteroidetes (F/B) ratio of each group was analyzed. The F/B ratio of the P group was higher than that of the C group. However, the Z group decreased significantly compared with the P group (**Figure 4C**). The above results suggest that ZDD can decrease Firmicutes, increase Bacteroidetes, and reduce the F/B ratio. In the case of PPC, the opposite effects were found.

At the genus level, Faecalibaculum, Bacteroides, Blautia, and Colidextribacter dominated the flora composition (**Figures 4D, E**). The Faecalibacterium and Bacteroidetes accounted for a large proportion in the C group, while Brautia and Colidextribacter accounted for a small proportion. Interestingly, the Z group had a similar proportion of the above four genera as the C group, and there was no statistical difference between the two groups (**Figure 4F**). The above results suggested that ZDD can make the intestinal flora of mice more inclined to those of healthy mice in the control group at the genus level.

#### 3.2.3 LEfSe differential analysis of intestinal flora

**Figure 5:**
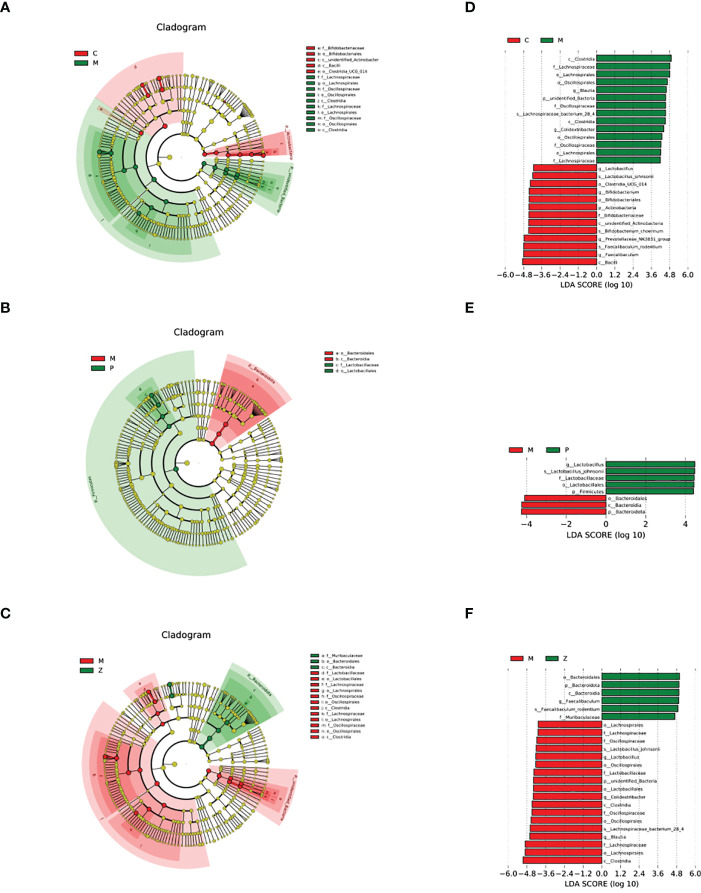
LEfSe differential analysis of intestinal flora. **(A)** Cladogram between C and M groups. **(B)** Cladogram between M and P groups. **(C)** Cladogram between M and Z groups. The order of the circles radiating from the inside to the outside in the cladogram is for each level (kingdom, phylum, class, order, family, genus, and species). The size of the diameter represents the size of the relative abundance. If the color in the small circle is yellow, it means that there is no significant difference in the level of bacteria; if the color is the color of the group, it means the bacteria with a significant difference in the group, and the corresponding flora name is displayed on the right side. The letter number corresponds to the figure. **(D)** LDA value histogram between C and M groups. **(E)** LDA value histogram between M and P groups. **(F)** LDA value histogram between M and Z groups. The color of the histogram in the LDA value histogram represents the group. The length represents the LDA score, i.e., the degree of influence of bacteria (groups) with significant differences between groups.

The LEfSe analysis with LDA=4 was used to examine differences in species composition. The results indicated that the M group differed from the C group due to the up-regulation of (Firmicutes - Clostridia - Lachnospirales - Lachnospiraceae - Blautia) and (Firmicutes - Clostridia - Oscillospirales - Oscillospiraceae - Colidextribacter) (**Figures 5A, D**). Interestingly, the difference between the M and Z groups is similar to the difference between the M and C groups (**Figures 5C, F**). The difference between the M and P groups was due to the up-regulation of Bacteroidota - Bacteroidia - Bacteroidales) (**Figures 5B, E**). The above results suggested that in LEfSe differential analysis, ZDD can make the intestinal flora of mice more similar to that of healthy mice in the control group.

### 3.3 The effect of ZDD on the intestinal SCFAs of NAFLD model mice

**Figure 6:**
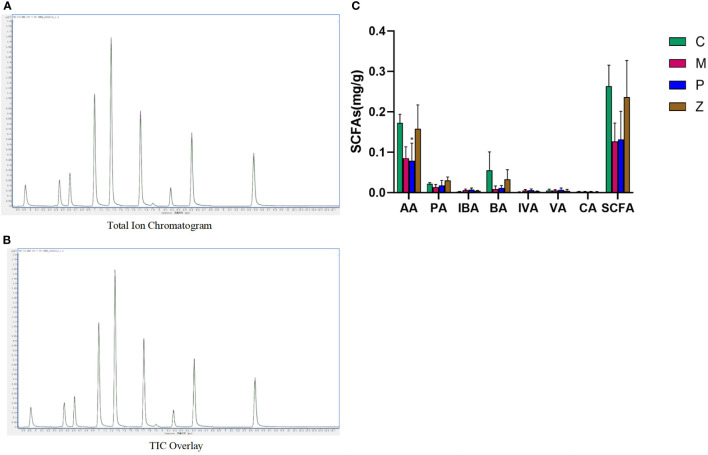
Intestinal short-chain fatty acids. **(A)** Total Ion Chromatogram. **(B)** TIC Overlay. **(C)** Content of various types of short-chain fatty acids. Data in histograms are expressed as mean ±SD. * is P < 0.05 compared with C group; ** is P < 0.01 compared with C group; # is P < 0.05 compared with M group; ## is P < 0.01 compared with M group; ^ is P < 0.05 compared with P group; ^^ is P < 0.01 compared with P group. TIC, total ion chromatogram; AA, acetic acid; PA, propionic acid; IBA, isobutyric acid; BA, butyric acid; IVA, isovaleric acid; VA, valeric acid; CA, caproic acid; SCFAs, short-chain fatty acids.

The linear equation and determination coefficient of the standard curve of the substances tested in this project are shown in **Table 2**. The sample quality control analysis results showed that the total ion chromatograms had high overlap, suggesting that the retention time and peak intensity were consistent, indicating that the instrument was stable during the detection of this project (**Figures 6A, B**). And these quantitative results which can indicate the stability of the instrument have been provided in the additional materials (Table file named Total Ion Chromatogram). The results showed that AA, PA, and BA dominated the composition of SCFAs. Interestingly, AA, PA, BA, and SCFA content in the Z and C groups maintained high levels (**Figure 6C** and **Supplementary Table 1**). This also indicates that ZDD can make the content and internal ratio of SCFAs in the gut more similar to that of healthy mice in the control group.

**Table 2:** Linear equations.

Index	Class	RT	Equation	R2	Weighting	LLOQ	ULOQ
AA	SCFA	5.918	y = 0.973708 x + 0.442955	0.9996124	1/x	0.02	20
PA	SCFA	6.448	y = 0.357051 x + 0.027106	0.999585212	1/x	0.02	20
IBA	SCFA	6.612	y = 1.606677 x	0.999867439	1/x	0.02	20
BA	SCFA	6.997	y = 10.035359 x + 0.163830	0.999793938	1/x	0.05	20
IVA	SCFA	7.25	y = 12.378349 x - 0.034708	0.999799192	1/x	0.05	20
VA	SCFA	7.706	y = 6.223082 x + 0.136725	0.999832054	1/x	0.05	20
CA	SCFA	8.502	y = 2.534667 x	0.999927206	1/x	0.05	20

Index is the name of the substance; the equation is linear; R2 is the coefficient of determination; weighting is the weight; LLOQ (μg/mL) and ULOQ (μg/mL) are the lower limits of quantification, and the upper limit of quantification, respectively can be accurately quantified.

### 3.4 The effect of ZDD on the ileal occludin and ZO-1 proteins of NAFLD model mice

**Figure 7:**
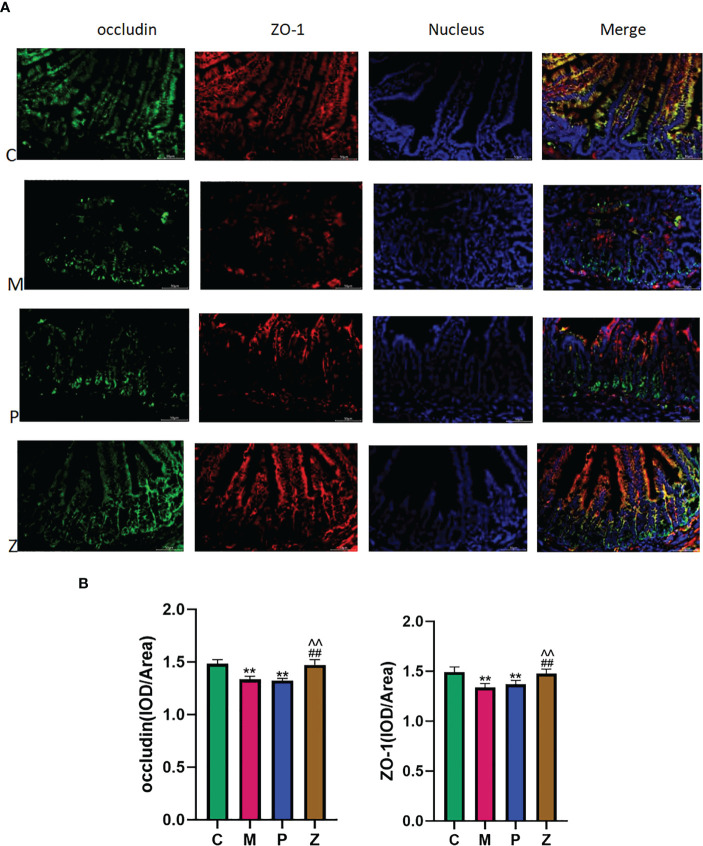
Intestinal short-chain fatty acids. **(A)** Expression and distribution of occludin and ZO-1 in ileal tissue (400x). **(B)** IOD/Area expression of occludin and ZO-1 in ileal tissue (Analyzed using Image-ProPlus 6.0 software). Data in histograms are expressed as mean+SD. * is P < 0.05 compared with C group; ** is P< 0.01 compared with C group; # is P< 0.05 compared with M group; ## is P < 0.01 compared with M group; ^ is P < 0.05 compared with P group; ^^is P < 0.01 compared with P group. IOD, Integrated Optical Density.

In terms of occludin, the M and P groups’ protein expression was significantly lower than the C group, with discontinuous aggregation and disordered arrangement (**Figure 7A**). Interestingly, the protein expression levels of the Z and C groups remained high, with no difference between them (**Figure 7B**). The situation of ZO-1 was similar to occludin. The above results indicated that ZDD could make the expression of TJ proteins occludin and ZO-1 between ileal mucosal epithelial cells more similar to that of healthy mice in the control group.

## 4 Discussion

### 4.1 Interpretations

We successfully established a NAFLD model with a 10-week HFD, characterized by increased body weight, disturbing serum liver function and lipid values, and abnormal liver pathology. Furthermore, we also found differences in the expression of intestinal flora, SCFAs, and TJ proteins in ileal mucosal epithelial cells between the M and C groups, which is a good hint for our study to investigate mechanisms in these areas. Furthermore, it was also confirmed that ZDD and PPC have different effects on NAFLD efficacy after four weeks of treatment. Despite having a different emphasis, the therapeutic mechanism of PPC does not seem to be related to the liver-gut axis as compared to ZDD. Because PPC did not alter intestinal flora, SCFAs, and TJ proteins between ileal mucosal epithelial cells. However, the role of ZDD in these aspects was prominent.

### 4.2 Intestinal flora

We analyzed the intestinal flora to explore further how ZDD exerts a therapeutic effect on NAFLD. Intestinal flora diversity analysis showed that ZDD could make the general composition of the intestinal flora more similar to that of healthy mice in the control group. Although there is no significant difference between Group M and Group C on the Chao 1 index and Shannon index expressing the community richness and diversity. After PCoA statistics, it was found that each group of samples had better intra-group aggregation. Though, there were differences with other groups of samples, indicating the pathogenesis of the disease and the treatment mechanism of intervention measures were closely related to the intestinal flora. The results obtained by the Amova algorithm also showed that the intestinal flora structure of the M group was significantly different from that of the C group. However, after ZDD intervention, the intestinal flora structure of the Z group was more similar to that of healthy mice in the control group. There was no statistical difference between C and Z groups. So far, we are more convinced of the need for further research based on the direction of intestinal flora.

#### 4.2.1 Intestinal flora at the phylum level

To further explore the internal changes of the flora structure, we conducted research at the phylum level, namely the similarity of the flora structure between Z and C groups and their difference with the M group. The results showed that ZDD could decrease Firmicutes, increase Bacteroidetes, and decrease the F/B ratio. Previous studies have shown that Firmicutes have a positive correlation with metabolic diseases. In contrast, Bacteroidetes have a negative correlation with it, and the decrease in their F/B ratio can reduce the body’s calorie intake from food, thereby avoiding obesity (Plovier et al., 2017; Ruiz et al., 2017; Sharpton et al., 2018).

#### 4.2.2 Intestinal flora at the genus level

To explore the changes in the flora below the phylum level, genus-level investigations were carried out. The results showed that ZDD could increase the proportion of Faecalibacterium and Bacteroidetes while decreasing the ratio of Brautia and Colidextribacter. The results were similar to that of the C group but opposite in the case of the M group. It is suggested that ZDD can alter the intestinal flora of mice to be more similar to that of healthy mice in the control group at the genus level. Previous studies have shown that Faecalibacterium can produce BA to improve mucosal barrier function, but its proportion in the gut of NASH and NAFLD patients is low (Wong et al., 2013; Da Silva et al., 2018). Furthermore, studies have revealed that Faecalibacterium can induce regulatory T cells to improve mucosal barrier function to treat NASH (Kessoku et al., 2018). In terms of Bacteroides, previous studies have shown that obesity indicators are negatively correlated with Bacteroides (Zeng et al., 2019). An increase in the proportion of Bacteroides is beneficial for optimizing serum liver function and blood lipids (Lang et al., 2020) and reducing hepatic fat fraction (Orabi et al., 2021). Furthermore, animal studies have also shown that Bacteroidetes have anti-obesity effects (Gauffin Cano et al., 2012; Fernández-Murga and Sanz, 2016). In terms of Blautia, existing studies have shown contradictory results, where Blautia has been reported to be positively correlated with BMI (Ottosson et al., 2018). Another study found that Blautia was negatively correlated with the visceral fat area and even helped reduce blood lipids and resist inflammation (Tong et al., 2018; Ozato et al., 2019). Since NAFLD is an intrahepatic inflammation, we speculate that the emergence of Blautia is more likely a result of inflammation. In terms of Colidextribacter, studies have reported that it is positively correlated with the degree of fibrosis in NAFLD (Hoyles et al., 2018; Aron-Wisnewsky et al., 2020). Colidextribacter can convert macrophage polarization to M1 phenotype and induce hepatocyte lipolysis, and the continuous occurrence of this process will accelerate the process of liver fibrosis (Zhang et al., 2020). Furthermore, Colidextribacter is the dominant bacteria of Gram-negative bacteria, and lipopolysaccharide (LPS), also known as endotoxin, which is the main component of the outer membrane of Gram-positive bacteria, is a major cause of endotoxemia (Cani et al., 2008). Chronic low-grade inflammation due to metabolic endotoxemia is considered an important marker of metabolic diseases such as obesity (Cani et al., 2007). So, the emergence of the above-discussed four genera and the pathogenesis incurred as a result are interrelated and are proven to be mutually supportive and consistent.

#### 4.2.3 Intestinal flora at LEfSe analysis level

Next, we used the LEfSe analysis method to determine which flora cause community differences, i.e., the core flora. We found that the reason why the M group differs from the Z group is quite similar to the reason the M group differs from the C group, which is up-regulation of (Firmicutes - Clostridia - Lachnospirales - Lachnospiraceae - Blautia) and (Firmicutes - Clostridia - Oscillospirales - Oscillospiraceae - Colidextribacter). Two conclusions can be drawn from this: First, the core flora in the NAFLD model is dominated by a high proportion of Firmicutes. Second, this phenomenon shows that ZDD can make the intestinal flora more similar to that of healthy mice in the control group.

### 4.3 SCFAs

As per the findings of many studies (Yu et al., 2016; Ji et al., 2019), changes in the intestinal flora will affect the expression of SCFAs, which can ultimately affect the permeability of the intestinal mucosa. Higher permeability will bring harmful substances such as pathogenic bacteria and their metabolites, like LPS, into the liver through the enterohepatic circulation, causing NAFLD. Next, we will detect intestinal SCFAs and TJ proteins between ileal mucosal epithelial cells to determine whether this mechanism is unobstructed and whether ZDD plays a role in treating diseases by participating in this mechanism.

The results of SCFAs revealed that AA, PA, and BA dominated SCFAs composition. Interestingly, AA, PA, BA, and SCFA contents in the Z and C groups were consistent with higher levels. SCFAs are organic compounds produced by the fermentation of carbohydrates by intestinal microbes. They are mainly composed of AA, PA, and BA, which account for 90% to 95% of the total SCFAs (Canfora et al., 2019). The AA and PA, in particular, can reduce excessive energy and fat deposition in the body by enhancing the release of glucagon-like peptide-1 (GLP-1) and peptide YY (PYY) to reduce appetite and increase satiety (Tolhurst et al., 2011; Larraufie et al., 2018). As BA can supply energy to intestinal epithelial cells, sufficient BA synthesis is the key to ensuring the full expression of intestinal transepithelial electrical resistance and TJ proteins, thereby reducing intestinal mucosa permeability (Barcenilla et al., 2000; Zhou and Fan, 2019; Ho Do et al., 2020). Moreover, BA is mainly produced by Bacteroides (Stumpff, 2018), and 16S rRNA sequencing showed that the Bacteroides contents in the C and Z groups were higher, which translated into higher BA contents in groups C and Z. However, there was no significant difference in the content of various SCFAs between the P and the M groups, suggesting that PPC had little effect on the intestinal SCFAs content. We were not surprised because the P group did not differ significantly from the M group in 16S rRNA sequencing.

Therefore, the detection of SCFAs played a linking role in the whole experiment. In 16S rRNA sequencing, ZDD was found to maintain the Z group’s intestinal flora structure similar to that of the C group. The upstream of the link was that detection of SCFAs verifies that similar intestinal flora can maintain a high similarity in the composition and content of SCFAs; for example, higher Bacteroides can produce higher BA. The downstream of the link is to observe whether the C and Z groups with high content of SCFAs and a high proportion of BA can reduce the passage of the intestinal mucosal mechanical barrier in the following Immunofluorescent detection.

### 4.4 Occluding and ZO-1 in intestinal mucosal mechanical barrier

Under normal physiological conditions, intestinal mucosal barrier function can prevent pathogenic bacteria and related metabolites in intestinal cavity from transferring out of intestinal cavity. The intestinal mucosal mechanical barrier is composed of intestinal epithelial cells, TJ, and the mucus layer covering the surface of the epithelial cells. As an important part of the intestinal mucosal mechanical barrier, TJ is located at the top of epithelial cells, surrounds the cells in a hoop shape, and tightly connects adjacent epithelial cells. And, TJ can strengthen the intestinal mucosal barrier function by reducing the permeability to prevent the translocation of pathogenic microorganisms and their metabolites from the intestinal tract into the portal venous system, causing chronic liver inflammation and eventually NAFLD. Therefore, the down-regulation and abnormal distribution of TJ protein are the main molecular mechanism of intestinal mucosal barrier dysfunction associated with NAFLD. As occludin and ZO-1 are important constituent TJ proteins, we performed immunofluorescence detection on occludin and ZO-1. The results showed that both the Z and C groups maintained good morphological expression and high expression of occludin and ZO-1. However, the situation in the M and P groups is not optimistic. This suggests that ZDD is responsible for maintaining the complete expression of occludin and ZO-1. Their full presentation is conducive to the normal functioning of the intestinal mucosal mechanical barrier, preventing pathogenic bacteria and their metabolites LPS from entering the liver through the portal vein and thereby preventing and treating NAFLD (Thanabalasuriar et al., 2013; Giorgio et al., 2014).

## 5 Conclusion

In conclusion, we confirmed the therapeutic effect of ZDD on NAFLD through animal experiments. We explored the mechanism of this effect in terms of intestinal flora, SCFAs, and intestinal mucosal mechanical barrier. Finally, we conclude that the therapeutic mechanism of ZDD may be the result of the synergy of the aspects mentioned above. However, fecal bacteria transplantation technology has gradually emerged in recent years. Unfortunately, the fecal bacteria transplantation, inflammatory markers and related pathways experiment was not performed in the current study to prove the above conclusions in reverse. As the technology matures, it will be supplemented in future experiments.

**Figure 8:**
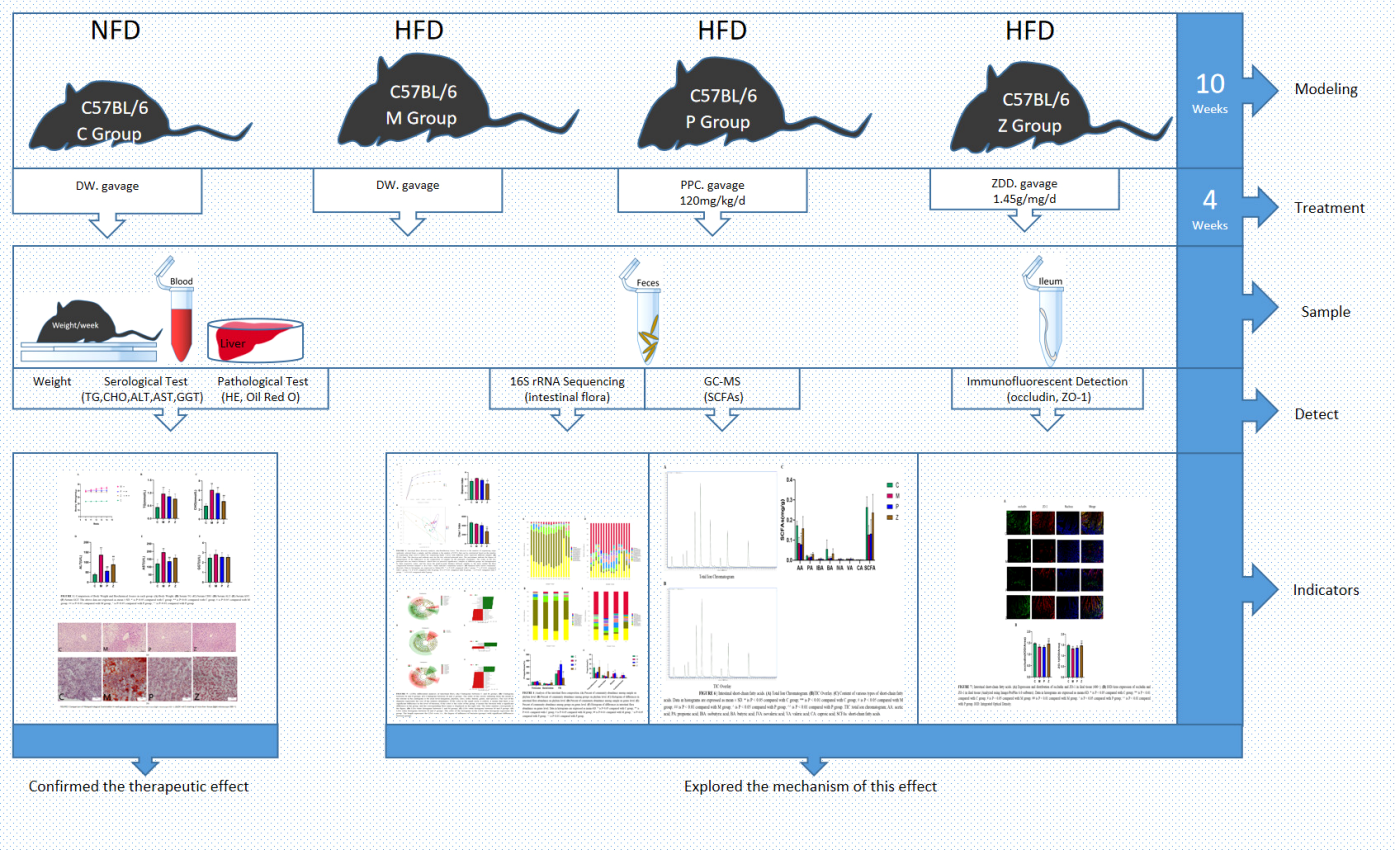
Experimental design diagram.

**Figure 9:**
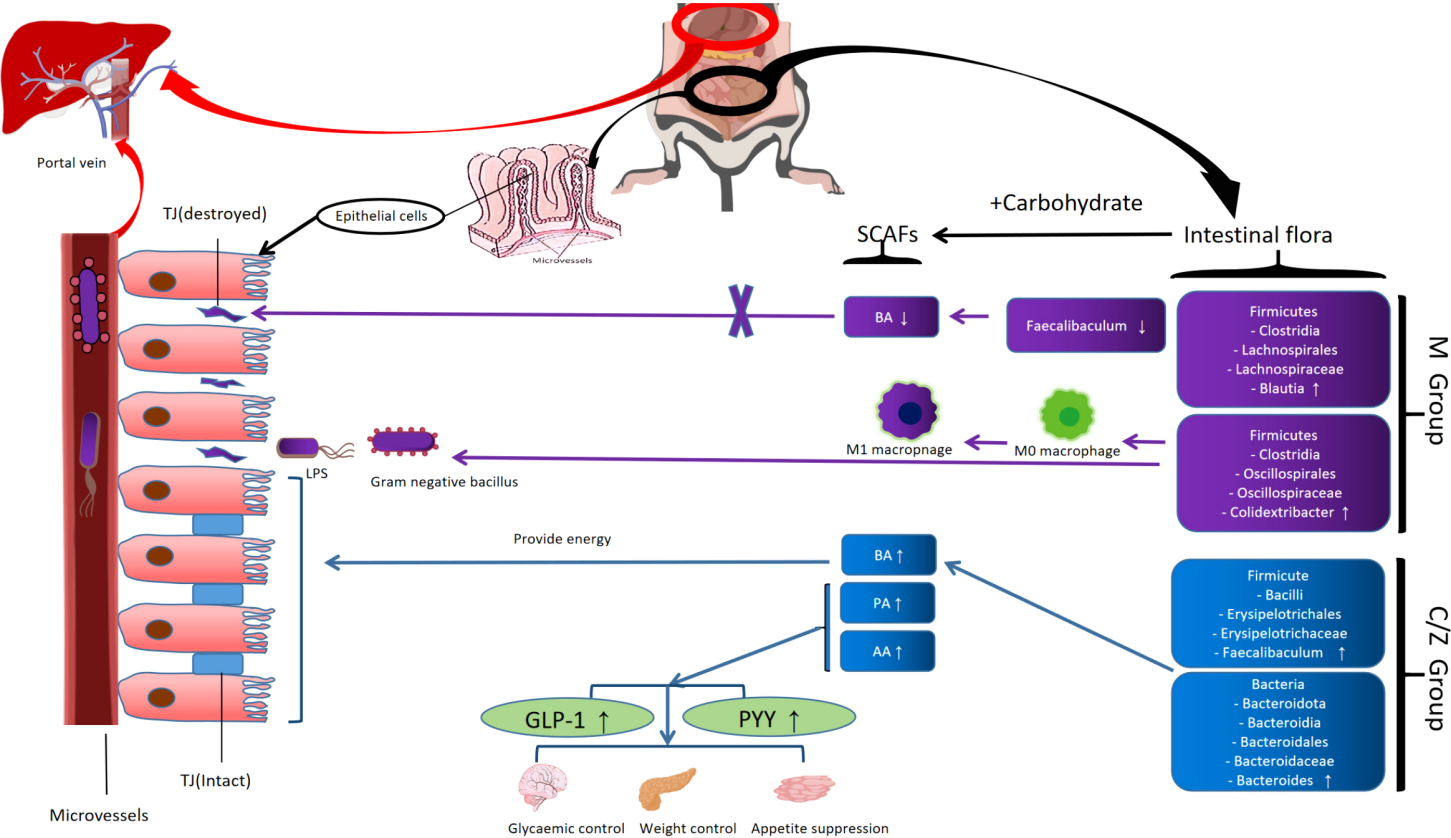
The mechanism of the ZDD's therapeutic effect for NAFLD based on intestinal flora.

Experimental design diagram (**Figure 8**); The mechanism of the ZDD’s therapeutic effect for NAFLD based on intestinal flora (**Figure 9**).

## Data availability statement

The datasets presented in this study can be found in online repositories. The names of the repository/repositories and accession number(s) can be found in the article/**Supplementary Material**.

## Ethics statement

The animal study was reviewed and approved by Animal Experiment Ethics Committee of the First Hospital of Jilin University.

## Author contributions

As the first author, CRB took in charge of the whole process of the experiments, and the writing and modification of this manuscript. JTS participated in the translation and dification of this manuscript and sorted all pictures. JD participated in the experiments and revised this manuscript. LYC, YJLi participated in the experiments and guided the writing. XYJ, YL, WPZ and YCL analyzed the data. YJLiu guided the experimental methods and directed the design of the project. All authors contributed to the article and approved the submitted version.
